# Photocleavable Regenerative Network Materials with Exceptional and Repeatable Viscoelastic Manipulability

**DOI:** 10.1002/advs.202101143

**Published:** 2021-08-02

**Authors:** Minami Oka, Hideaki Takagi, Tomotaka Miyazawa, Robert M. Waymouth, Satoshi Honda

**Affiliations:** ^1^ Department of Basic Science Graduate School of Arts and Sciences The University of Tokyo 3‐8‐1 Komaba Meguro Tokyo 153‐8902 Japan; ^2^ Photon Factory Institute of Materials Structure Science High Energy Accelerator Research Organization 1‐1 Oho Tsukuba Ibaraki 305‐0801 Japan; ^3^ Department of Materials and Engineering School of Materials and Chemical Technology Tokyo Institute of Technology 2‐12‐1 S8‐7 Ookayama Meguro‐ku Tokyo 152‐8552 Japan; ^4^ Department of Chemistry Stanford University Stanford CA 94305 USA

**Keywords:** mechanical properties, network materials, poly(dimethyl siloxane)s, solventless photocontrol

## Abstract

The development of solventless system for modulating properties of network materials is imperative for the next generation sustainable technology. Utilization of photostimulation is important owing to its spatial and temporal locality, yet designing photoresponsive network materials exhibiting repeatable and dramatic change in their properties remains a challenge. Here, the authors report a photocleavable regenerative network (PRN) linked with photoresponsive hexaarylbiimidazoles (HABIs) synthesized from narrow dispersity star‐shaped poly(dimethylsiloxane)s (PDMSs) having 2,4,5‐triphenylimidazole end groups. The use of urea anion as a catalyst for ring opening polymerization (ROP) of cyclic siloxane initiated from silanols enables control of molecular weight and dispersity. The rheological measurements for the synthesized PRNs exhibit drastic changes in storage and loss moduli (*G*′ and *G*″) upon photoirradiation in the solid state (*G*′ > *G*″). This photocontrolled change in viscoelasticity with retaining solidity enables application of PRNs as a remotely‐controlled photo‐melt adhesive and photo‐scissible string. The developed PRNs will enable a wide variety of applications such as industrially important next‐generation sustainable adhesive, sealant, and reversibly‐deformable 3D printing materials with their spatially and temporally local manipulability, solventless handleability, and excellent reversibility.

## Graphical abstract

Photocleavable regenerative networks (PRNs) are synthesized from structurally precise star‐shaped poly(dimethyl siloxane) precursors to realize a solventless spatially and temporally local dramatic change in mechanical properties. The photocontrolled dynamic change in viscoelasticity in the solid state enables application of PRNs as solventless remotely‐controlled photo‐melt adhesive and photo‐scissible string.

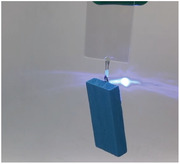

## 1 Introduction

**Figure 1:**
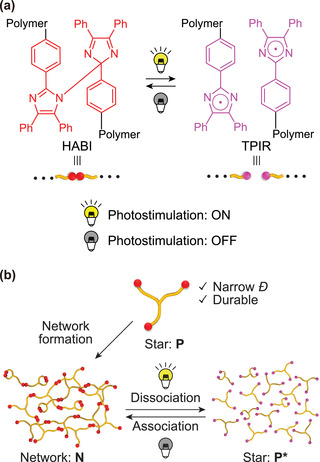
a) Photochemistry of HABI. b) Schematic illustration of photocleavable regenerative network (PRN).

Moldability of polymeric materials undoubtedly contributes to our lives. The soft and viscoelastic nature of polymeric materials enables the formation of various complex shapes. Thermal stimulation has generally been applied for controlling the viscoelasticity of materials, yet expansion of available stimulation is highly desirable.^[^ ^1^ ^]^ The spatiotemporal control possible with focused photostimulation is particularly attractive as a means of modulating the local viscoelasticity of materials. Recently, polyacrylates and polymethacrylates with azobenzene side chains have been shown to change their viscoelasticity upon photoirradiation, relying on the photoisomerization of azobenzenes on side chains.^[^ ^2^ ^]^ The glass transition temperatures (*T*_g_s) of polymers with *trans*‐isomeric side chains were higher than room temperature; *trans* to *cis* photoisomerization of side chains upon photoirradiation lowered the *T*_g_s below room temperature, attaining repeatable change in mechanical properties with the same polymers without solvent. On the other hand, we have synthesized a photocleavable regenerative network (PRN) composed of poly(dimethylsiloxane) (PDMS) linked with photoresponsive hexaarylbiimidazoles (HABIs) in the chains from the corresponding star‐shaped precursors with 2,4,5‐triphenylimidazole (lophine) end groups.^[^ ^3^ ^]^ Upon photoirradiation, HABI generates a pair of triphenylimidazoryl radicals (TPIRs) and the generated TPIRs undergo recoupling into HABI again by terminating photoirradiation (**Figure** **1**a).^[^ ^4^ ^]^ When elevated temperature to 100 °C, TPIRs produce non‐photoresponsive isomers of HABI that are not photoresponsive,^[^ ^5^, ^6^ ^]^ yet this reversible photoreaction proceeds quantitatively at lower temperatures. Owing to the stability of TPIR even under the presence of oxygen,^[^ ^7^ ^]^ solventless network scission and reformation at room temperature was realized in air; nevertheless, for these very soft materials, the loss modulus (*G*″) remained higher than storage modulus (*G*′) irrespective of photoirradiation.^[^ ^3^ ^]^ As more uniform networks are known to have superior mechanical properties,^[^ ^8^ ^]^ we anticipated that better control of molecular weight (MW) and dispersity of star‐shaped precursors for the network would lead to stiffer networks and more dramatic changes in viscoelasticity upon photoirradiation.

Herein we show that a urea anion‐catalyzed ring opening polymerization (ROP) of cyclic siloxanes initiated from a trifunctional arylsilane core affords star‐shaped PDMSs with narrow dispersity that can be capped with HABIs to generate well‐defined networks that show dramatic changes in their properties upon photoirradiation (Figure 1b). The combination of silanol initiators and a urea anion catalyst^[^ ^9^ ^]^ for ROP of hexamethylcyclotrisiloxane (D3) enabled the controlled synthesis of PDMSs with narrow dispersity (*Đ* < 1.1). The subsequent introduction of lophines and oxidation of lophines into TPIRs and their spontaneous coupling into HABIs produced network PDMSs functionalized with HABIs in the chains. Rheological studies revealed dramatic and reversible changes in viscoelasticity upon photoirradiation in the solid state. By taking advantage of properties of the present PRN, we further succeeded in applying our PRN as a remotely‐controlled photo‐melt adhesive and photo‐scissible string.

## 2 Results and Discussion

To identify the optimal conditions for generating well‐defined polysiloxanes, we carried out model reactions using dimethylphenylsilanol as an initiator for ROP of D3 with either 1,5,7‐triazabicyclo[4.4.0]dec‐5‐ene (TBD)^[^ ^3^, ^10^ ^]^ or urea anions^[^ ^9^ ^]^ as catalysts. These studies revealed that the urea anion derived from 1,3‐bis(3,5‐bis(trifluoromethyl)phenyl)urea, U(4CF_3_) and NaH afforded narrow molecular weights and the optimal reaction rates for polymerizations carried out in batch (Figure S1a, Supporting Information); the ROP of D3 was completed within 30 min (Figure S1b, Supporting Information). Although broadening of dispersity was observed after the 30 min due to equilibrium polymerization, size exclusion chromatography (SEC) showed unimodal traces (Figure S1b, Supporting Information) with narrow dispersities (*Đ* < 1.2) at monomer conversions ≤63% (Figure S1c, Supporting Information).

**Figure 2:**
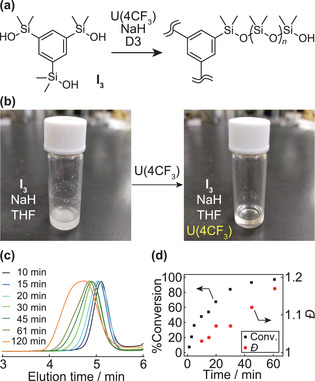
a) ROP of D3 initiated from **I_3_** with U(4CF_3_). b) Photos of a mixture prepared from **I_3_**, NaH, and THF before (left) and after (right) adding U(4CF_3_). c) SEC traces of products after ROP at 10, 15, 20, 30, 45, 61, and 120 min, respectively. d) Plots of conversion and dispersity against time.

Next, ROP of D3 initiated from trifunctional silanol (**I_3_**) was investigated to generate star polysiloxanes (**Figure** **2**a). The trisilanol **I_3_** exhibits relatively poor solubility in THF; deprotonation of **I_3_** with NaH in THF resulted in the formation of large precipitates (Figure 2b, left). Nevertheless, addition of U(4CF_3_) to this mixture solubilized the ionic species to give a homogeneous solution (Figure 2b, right). SEC monitored through the following ROP of D3 showed unimodal traces having narrow dispersities for conversions ≤80% (≈40 min, Figure 2c,d). The conversion of D3 reached 96% at 60 min although the dispersities increase slightly (Figure 2d). The urea anion catalysts are particularly useful for generating the lower molecular weight polysiloxane stars (Table S1, Supporting Information, entry 1–4). Moreover, the combination of silanols and urea anion catalysts overcomes the solubility problem derived from mismatch of polarity between initiators, D3, and PDMS. On the other hand, TBD is less effective at solubilizing the **I_3_** initiator and leads to polysiloxanes of broader polydispersities, even for the lower molecular weights (*Đ* = 1.34, *M*_n_ = 9200 Da); however, TBD enabled production of high‐MW PDMS (*Đ* = 1.35, *M*_n_ = 63 300 Da).^[^ ^10^ ^]^

**Figure 3:**
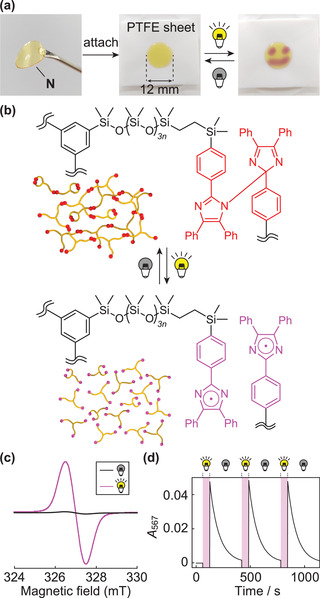
a) Photos of a film prepared from **N_1_** (left) without (middle) and with (right) photoirradiation on the PTFE sheet (*λ* ≈410 nm). b) Change in the chemical structure through network cleavage and reformation upon photoirradiation. c) ESR spectra of **N_1_** before (left) and after (right) photoirradiation (*λ* ≈410 nm). d) Time‐dependent plots of A_567_ upon ON–OFF of UV irradiation cycles. The sample was irradiated during time ranges indicated in pink (*λ* = 365 nm).

Having established the control of ROP of D3, we synthesized star‐shaped PDMSs with lophine end groups (**P**) by the end capping reaction with chlorodimethylvinylsilane and subsequent hydrosilylation with hydrosilane‐appended lophine according to the reported procedure (Scheme S1, Supporting Information).^[^ ^11^ ^]^ Oxidation of lophine end groups into TPIRs and their spontaneous coupling into HABI formed the corresponding network PDMSs (**N**s), that is, PRNs linked with HABIs in the chains (Scheme S1, Supporting Information, bottom). All of these PDMSs were characterized based on ^1^H NMR (Figure S2, Supporting Information) and SEC (Table S2, Supporting Information). The networks **N_1_** and **N_2_**, derived from **P_1_** (*M*_n_ = 7400 Da) and **P_2_** (*M*_n_ = 11 200 Da), were transparent yellowish films (**Figure** **3**a, left) and showed response to photostimulation only at the irradiated region to change the color into pink (Figure 3a, middle and right), suggesting progress of the intended photochemistry of HABI within the material (Figure 1a). The physical properties of the networks **N** depend on the molecular weight of the precursors **P**. Whereas networks derived from **P_1_** and **P_2_** are stable films, the **N_3_**, derived from the higher molecular weight star **P_3_** (*M*_n_ = 50 400 Da) is a yellowish liquid. Differences in topological inhomogeneity^[^ ^12^ ^]^ between the networks likely affected their physical states. That is, most of the reactions between TPIRs for the higher molecular weight materials were intramolecular and thus it was difficult to construct a long‐range network.

The transparency of **N** also suggests the inner homogeneity of the material. This is favorable because the formation of aggregates derived from moieties immiscible to polymer chains causes phase separation and would restrain change in the physical properties of PRNs upon photoirradiation. The nanoscale homogeneity of **N_2_** was validated by small‐angle X‐ray scattering (SAXS) analyses. The SAXS profile of **P_2_** showed an apparent scattering derived from aggregates of end groups (Figure S3, Supporting Information, blue line). This scattering pattern is similar to that that appeared for linear PDMSs with lophine end groups. As imidazoles form several types of H‐bondings,^[^ ^13^ ^]^ the end groups were probably aggregated within the material by H‐bonding between the imidazoles in lophines similar to a previous report.^[^ ^11^ ^]^ However, only a slight broad scattering derived from correlation hole appeared at the higher *q* region in the profile of **N_2_** (Figure S3, Supporting Information, red line). Therefore, the formation of aggregates was inhibited upon forming HABI and the SAXS profile revealed nanoscale homogeneity of **N_2_**. To further validate the formation of network structures within the materials, gels were prepared by swelling **N_2_** either with hexane or EtOAc and were subjected to SAXS analyses, expecting enhanced contrast of scattering between polymer chain and solvent. Both of SAXS profiles of gels from **N_2_** showed upward shift of the traces at Log *q* range roughly between −0.5 and 0 at the concentration of 5 and 10 wt% for hexane (Figure S4a, Supporting Information) and with at the concentration of 5 wt% for EtOAc (Figure S4b, Supporting Information). The scatterings appeared in these profiles are apparently different from those of end group aggregation (Figure S3, Supporting Information) but are in good agreement with those of gels formed from star‐shaped polymer networks.^[^ ^14^ ^]^ This strongly suggests the construction of the intended covalent bonding network within our PRNs.

The dynamic properties of **N**s upon photoirradiation are intriguing, because the network cleavage and rebonding can occur even in the solid state **N**s (Figure 3b). The formation of TPIRs upon photoirradiation was validated by comparing ESR spectra of **N_1_** before (Figure 3c, black line) and after photoirradiation (Figure 3c, pink line) by the appearance of the signal at 327 mT, which is identical to the previous report.^[^ ^6^ ^]^ A slight ESR signal appeared before photoirradiation indicates the presence of unreacted TPIRs due to the restricted mobility of polymer chains after forming the network (Figure 3c, black line). Effective consumption of the generated TPIRs upon photoirradiation has been validated by measuring ESR spectra over time, demonstrating that the almost complete disappearance of the ESR signal upon photoirradiation after 20 min (Figure S5, Supporting Information). Upon photoirradiation, the UV–vis spectrum of **N_1_** in the film state showed TPIR‐derived strong adsorption at the wavelength of 567 nm (*A*_567_) (Figure S6, Supporting Information). Time‐dependent plots of *A*_567_ during ON–OFF photoirradiation cycles showed repeated increase and decrease of *A*_567_ (Figure 3d), demonstrating reversibility of the photoreaction.

**Figure 4:**
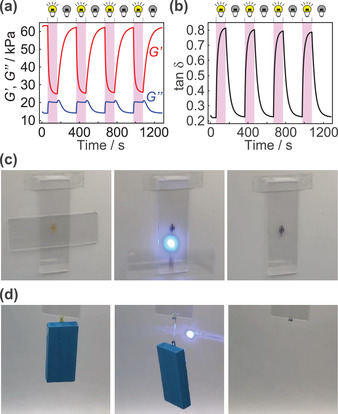
Time‐dependent plots of a) *G*′ and *G*″ and b) tan *δ* ( = *G*″/*G*′) upon ON–OFF of photoirradiation cycles to N_1_. The sample was irradiated during time ranges indicated in pink (*λ *= 365 nm). c) Photographs of glass slides attached on a wall by the N_1_ adhesive before (left), during (middle), and after (right) photoirradiation (*λ *≈410 nm) captured from Movie S1, Supporting Information. d) Photographs of glass slides connected with the wood brick with the N_1_ string before (left), during (middle), and after (right) photoirradiation (*λ *≈410 nm) captured from Movie S2, Supporting Information.

We have then performed rheological analyses of the film of **N_1_** upon ON–OFF photoirradiation cycles under solvent‐free isothermal conditions at room temperature (25 °C). From time‐dependent rheological analyses upon ON–OFF photoirradiation cycles, the storage modulus *G*′ showed a dramatic decrease from 65 to 25 kPa, whereas the loss modulus *G*″ increased from 15 to 20 kPa (**Figure** **4**a). Both of decrease in *G*′ and the increase in *G*″ are rapid (within 1 and 4 min, respectively), demonstrating significantly faster photoresponse of **N_1_** compared to the networks described in our previous study.^[^ ^3^ ^]^ The change in the viscoelasticity of **N_1_** upon photoirradiation can be evaluated by time‐course plots of loss tangent (tan *δ* = *G*″/*G*′), where the tan *δ* increased from 0.25 to almost 0.8 (Figure 4b). Given that the boundary of solid and liquid is often considered by the tan *δ*  of 1 (*G*′ = *G*″),^[^ ^15^ ^]^ these data indicate that photoirradiation induces a hard‐to‐soft transition of the network in the solid state (*G*′ > *G*″). Network **N_2_**_,_ derived from the higher molecular weight precursor **P_2_**, showed a photoresponse similar to that of **N_1_** (Figure S7a, Supporting Information), yet the magnitude of the change was less prominent. For **N_3_**, derived from the highest MW precursor **P_3_** (*M*_n_ = 50 400 Da) the response of *G*′ and *G*″ to photoirradiation was very small and the material maintained liquid state (*G*′ < *G*″) irrespective of photoirradiation (Figure S7b, Supporting Information).

We finally applied our PRNs as a remotely‐controlled photo‐melt adhesive (Movie S1, Supporting Information) and photo‐scissible string (Movie S2, Supporting Information) by taking advantage of the dramatic change in rheological properties upon photoirradiation. Thus, **N_1_** was sandwiched between two glass slides and the set of slides was attached on the wall (Figure 4c, left). Adherence property of **N_1_** was high enough to maintain the attached state at least for 2 weeks in the experimental period. Photoirradiation caused a loss of adhesion of the network **N_1_**, as the glass slide gradually slid down the plate (Figure 4c, middle) and finally detached (Figure 4c, right). Evidently, the increase of tan *δ* upon photoirradiation to **N_1_** led to the loss of adhesivity. Further, a wood brick was connected with a glass with a **N_1_** string (Figure 4d, left). Photoirradiation to the center of the string resulted in elongation (Figure 4d, middle) followed by scission (Figure 4d, right). It should be noted that these changes were achieved by a consumer usage blue laser pointer (*λ* ∼ 410 nm).

## 3 Conclusions

In summary, we have succeeded in synthesizing PRNs linked with HABIs from star‐shaped PDMSs with narrow dispersity. The ROP of D3 based on the combination of trifunctional silanol and urea anion enabled control of MW and dispersity. The rheological measurements for the PRNs disclosed that the drastic changes in *G*′ and *G*″ upon photoirradiation. We further demonstrated that this photocontrolled change in viscoelasticity with retaining solidity enables utilization of PRNs as the remotely‐controlled photo‐melt adhesive and photo‐scissible string. With their spatially and temporally local manipulability, solventless handleability, and excellent reversibility, our PRNs may find wide applications such as industrially important next‐generation sustainable adhesive, sealant, and reversibly‐deformable 3D printing materials.

## 4 Experimental Section

### ROP of D3 Catalyzed by Urea Anion

Into a flask was prepared a mixture of U(4CF_3_) (145 mg, 0.30 mmol), NaH (7.2 mg, 0.30 mmol), and THF (0.80 mL). To this mixture, **I_3_** (10 mg, 0.10 mmol for OH groups) was added and shook until a homogeneous solution was obtained. A separately prepared THF solution (3 mL) of D3 (2.0 g, 9.0 mmol) was added to the mixture and the resulting solution was stirred at room temperature (25 °C). After 12 min, THF solution (0.50 mL) of benzoic acid (122 mg, 1.0 mmol) was added and the mixture was further stirred for 120 min. An excess amount MeCN was added to the reaction mixture to cause phase separation into PDMS and MeCN layers. The PDMS layer was washed successively with MeCN and MeOH, and dried under reduced pressure to afford a hydroxy‐terminated three‐armed star‐shaped PDMS, which was used for the subsequent end‐capping reaction without further purification. Thus, to this PDMS, THF (3 mL), pyridine (480 µL, 6.0 mmol), and chlorodimethylvinylsilane (270 µL, 2.0 mmol) was added in this order and the mixture was further stirred for 21 h. The mixture was poured into an excess amount of H_2_O/hexane and aqueous layer was separated and extracted with hexane. The combined hexane phase was washed with water, dried over Na_2_SO_4_, and concentrated to dryness. The obtained crude oily product was washed with MeOH and dried under reduced pressure to afford vinyl‐terminated three‐armed star‐shaped PDMS (**S_1_**) as a colorless oil. The yield was 540 mg. ^1^H NMR (500 MHz, acetone‐*d*_6_, *δ*): −0.07–0.16 (—SiO(C*H*_3_)_2_—), 0.18 (s, —Si(C*H*_3_)_2_CH═CH_2_), 0.40 (ArSi(C*H*_3_)_2_O—), 5.79 (dd, —Si(CH_3_)_2_CH═C*H*_2_), 5.96 (dd, —Si(CH_3_)_2_CH═C*H*_2_), 6.17 (dd, —Si(CH_3_)_2_C*H*═CH_2_), 7.87 (s, Ar*H*).

### ROP of D3 Catalyzed by TBD

Into a flask was prepared a mixture of **I_3_** (300 mg, 3.0 mmol for OH groups) and TBD (139 mg, 1.0 mmol) in THF (2 mL). A separately prepared THF solution (20 mL) of D3 (11.1 g, 50 mmol) was added to the mixture and the resulting solution was stirred at 25 °C. After 80 min, pyridine (3.6 mL, 45 mmol) and chlorodimethylvinylsilane (4.1 mL, 30 mmol) was added in this order and the mixture was further stirred for 30 min. The mixture was poured into an excess amount of H_2_O/hexane and aqueous layer was separated and extracted with hexane. The combined hexane phases were washed with water, dried over MgSO_4_, and concentrated to dryness. The obtained crude oily product was washed with acetone and dissolved in CHCl_3_. The resulting solution was concentrated and dried under reduced pressure to afford **S_2_** as a colorless oil. The yield was 7.85 g. Synthesis of **S_3_** was performed in a similar procedure and both of **S_2_** and **S_3_** were characterized by ^1^H NMR in similar manners as **S_1_**.

### Hydrosilylation

In a typical procedure, a mixture of **S_1_** (450 mg, 0.21 mmol for vinyl groups), 2‐(4‐(dimethylsilyl)phenyl)‐4,5‐diphenyl‐1*H*‐imidazole (218 mg, 0.614 mmol), Karstedt's catalyst (0.10 mL), and THF (2.5 mL) was prepared in a flask and refluxed for 17 h. After cooling to room temperature, the mixture was concentrated to dryness. The residue was diluted with chloroform and poured into an excess amount of cold MeOH (−40 °C) to cause phase separation into PDMS and MeOH layers. The PDMS layer was diluted with a minimum amount of chloroform and concentration to dryness to afford three‐armed star‐shaped PDMS having lophine end groups (**P_1_**) as white solid. The yield was 240 mg (47%). ^1^H NMR (500 MHz, acetone‐*d*_6_, *δ*): −0.07–0.16 (—SiO(C*H*_3_)_2_—), 0.31 (—Si (C*H*_3_)_2_CH_2_CH_2_—), 0.40 (ArSi(C*H*_3_)_2_O—), 0.53 (—Si (CH_3_)_2_C*H*_2_CH_2_—), 0.78 (—Si (CH_3_)_2_CH_2_C*H*_2_—), 7.20–8.15 (Ar*H*), 11.72 (br, —N*H*). Likewise, **P_2_** and **P_3_** were synthesized from **S_2_** and **S_3_**, respectively.

### Oxidation of Lophines into TPIRs and their Spontaneous Coupling into HABIs

In a typical procedure, into a flask was prepared hexane (100 mL), **P_1_** (200 mg, 8.1 µmol for lophine end groups), and an aqueous solution (60 mL) containing potassium ferricyanide (1.33 g, 4.0 mmol) and potassium hydroxide (208 mg, 4.0 mmol) was added under dark. The mixture was vigorously stirred at room temperature for 2.5 h. The aqueous layer was separated and extracted with hexane. The combined hexane phases were washed with water, dried over Na_2_SO_4_, and evaporated. After drying under reduced pressure, network PDMS having HABIs in the chains (**N_1_**) was obtained as a transparent yellowish film‐like solid. The yield was 193 mg (97%). ^1^H NMR (500 MHz, acetone‐*d*_6_, *δ*): −0.07–0.31 (—SiO(C*H*_3_)_2_—, —Si (C*H*_3_)_2_CH_2_CH_2_—), 0.40 (ArSi(C*H*_3_)_2_O—), 0.45–0.58 (—Si (CH_3_)_2_C*H*_2_CH_2_—), 0.66–0.79 (—Si (CH_3_)_2_CH_2_C*H*_2_—), 7.00–7.90 (Ar*H*). Likewise, **N_2_** and **N_3_** were synthesized.

### Photoirradiation Experiments

Photoirradiation tests were carried out using an Asahi Spectra MAX‐350 xenon lamp equipped with an Asahi Spectra quartz light guide. An Asahi Spectra LX0365 bandpass filter was used for transmitting only the wavelength of 365 nm with the 80% intensity. For rheological analysis, photoirradiation was performed on a Hamamatsu LIGHTNING CURE LC8 L9588 model with a Hamamatsu A10014‐35‐0110 light guide and a Hamamatsu A9616‐07 filter was used for transmitting UV with the wavelength at around 365 nm (150 mW cm^−2^). For demonstrations (Figure 4c,d and Movies S1 and S2, Supporting Information), a consumer use blue laser pointer (*λ* ≈410 nm) was used.

### ESR Measurements

ESR spectra were recorded on a JEOL JES‐TE3000 spectrometer at room temperature. For photoirradiation experiments, photoirradiation was performed from a window equipped with a sample insertion opening.

### UV–Vis Absorbance Measurements

UV–vis absorption spectra were recorded on a SHIMADZU UV‐1900i spectrometer using a film holder accessory. For UV–vis absorbance measurements under photoirradiation, the light guide of the light source was set inside the sample chamber and photoirradiation was performed directly to the film sample.

### Rheological Analyses

Rheological analyses were performed on an Anton Paar MCR 102 rheometer equipped with a UV curing system and a Peltier temperature control device. Time‐dependent analyses of *G*′ and *G*″ for the thin film samples with the thickness of 0.3 mm were conducted using a parallel plate with a diameter of 12 mm at the frequency of 5 Hz at 25 °C.

## Conflict of Interest

The authors declare no conflict of interest.

## Supporting information

Supporting Information

Supplemental Movie 1

Supplemental Movie 2

## Data Availability

The data that support the findings of this study are available from the corresponding author upon reasonable request.
