# Supplementary material for: Photocleavable Regenerative Network Materials with Exceptional and Repeatable Viscoelastic Manipulability
Source: Adv Sci (Weinh). 2021 Aug 2;8(19):2101143. doi: 10.1002/advs.202101143 (PMC8498910; doi:10.1002/advs.202101143)
Supplement: Supplementary file 1 — Supporting Information [file ADVS-8-2101143-s003.pdf]

WILEY-VCH

© 2021 Wiley-VCH GmbH

ADVANCED SCIENCE
Open Access

Supporting Information

for *Adv. Sci.*, DOI: 10.1002/advs.202101143

Photocleavable Regenerative Network Materials with Exceptional and Repeatable Viscoelastic Manipulability

*Minami Oka, Hideaki Takagi, Tomotaka Miyazawa, Robert M. Waymouth, and Satoshi Honda**

Supporting Information

Photocleavable Regenerative Network Materials with Exceptional and Repeatable Viscoelastic Manipulability

*Minami Oka, Hideaki Takagi, Tomotaka Miyazawa,[c] Robert M. Waymouth,[d] and Satoshi Honda[a]**

Materials. 1,3,5-Tribromobenzene (98%, TCI), Mg (Wako), chlorodimethylsilane (95%, TCI), NaOMe (95%, Wako), benzil (99%, TCI), 4-bromobenzaldehyde (97%, TCI), ammonium acetate (97%, TCI), hexamethylcyclotrisiloxane (D3) (98%, Aldrich), 1,5,7-triazabicyclo[4.4.0]dec-5-ene (TBD) (98%, Aldrich), chlorodimethylvinylsilane (98%, Aldrich), platinum(0)-1,3-divinyltetramethyldisiloxane complex solution (Karstedt's catalyst) (Pt~2% in xylene, Aldrich), potassium ferricyanide (98%, Wako), potassium hydroxide (85%, Wako) and other reagents were used as received. 2-(4-(Dimethylsilyl)phenyl)-4,5-diphenyl-1*H*-imidazole[1] and 1,3-bis(3,5-bis(trifluoromethyl)phenyl)urea, U(4CF_3),[2] were synthesized according to the reported procedures.

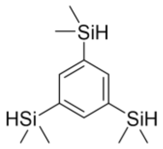

Synthesis of 1,3,5-tris(dimethylsilyl)benzene. Into a flask, tetrahydrofuran (THF) (100 mL), Mg (5.2 g, 0.214 mol), and chlorodimethylsilane (28.5 mL, 0.257 mol) were added. THF solution (100 mL) of 1,3,5-tribromobenzene (15.0 g, 0.143 mmol for Br groups) was slowly added to the flask and the reaction mixture was refluxed for 4 h. After cooling to room temperature, the mixture was filtered and the filtrate was concentrated to dryness. A hexane soluble part of the residue was collected, washed with H_2O, dried over $MgSO_4$. After evaporating the hexane, 1,3,5-tris(dimethylsilyl)benzene was obtained as an oily liquid. The yield was 7.26 g (60%). ^{1}H NMR (500 MHz, $CDCl_3$, δ): 0.37 (d, 18H, –Si(CH_3)$_2$H) 4.44 (sept., 3H, –Si(CH_3)$_2$*H*), 7.74 (s, 3H, Ar*H*).

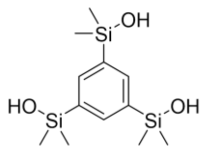

Synthesis of I_3. Into a flask, 1,3,5-tris(dimethylsilyl)benzene (7.26 g, 86.2 mmol for –SiH groups) and EtOH (10 mL), NaOMe (54 mg, 1 mmol) was added and the mixture was stirred at 70 °C for 2.5 h. After cooling the reaction mixture with an ice–water bath, a mixture of NaOH (34.5 g, 0.862 mmol), MeOH (100 mL), and H_2O (50 mL) was added. After further stirring for 30 min, H_2O (150 mL) was added and the resulting mixture was poured into aqueous solution (100 mL) of KH_2PO_4 (153 g, 1.12 mol) containing ice (ca. 100 g). After filtration, the solid collected was dissolved in ether, washed with H_2O, drying over $MgSO_4$, and concentrated to crystallize I_3 as a white solid. The yield was 3.65 g (42%). ^{1}H NMR (500 MHz, acetone-d_6, δ): 0.31 (s, 18H, –Si(CH_3)$_2$OH) 4.83 (s, 3H, –OH), 7.89 (s, 3H, ArH).

NMR measurements. ^{1}H NMR spectra were recorded on a Bruker AVANCE III spectrometer operating at 500 MHz. $CDCl_3$ or DMSO-d_6 was used as the solvent and chemical shifts were reported relative to tetramethylsilane (TMS) (δ = 0.00 ppm) or solvent residual signals.

SEC measurements. SEC measurements were performed using a Waters e-2695 high-speed liquid chromatograph equipped with RI and UV detectors. A Shodex KF-603 column (flow rate: 0.50 mL min^{-1}) or two series-connected TSKgel SuperMultipore HZ-H columns (flow rate: 0.35 mL min^{-1}) were employed with THF as the eluent at 40 ^{o}C.

Synchrotron radiation (SR) small angle X-ray scattering (SAXS) measurements. The SR-SAXS measurements were performed at the BL-10C beamline in KEK-PF at Tsukuba, Japan or at the BL19B2 in SPring-8 at Hyogo, Japan. At both beamline, PILATUS3 2M (DECTRIS) was commonly used as a detector and the calibration was carried out by measuring scattering vector $q = 4\pi \sin\theta/\lambda$ of silver behenate as a standard, where λ is the wavelength of x-ray, and the two-dimensional scattering images were circularly averaged to convert to one-dimensional data of scattering intensity against q by using data processing

software SAngler or Plot Radially. At BL-10C in KEK-PF, λ was 1.0 Å, the camera length was 3 m. At BL19B2 in SPring-8, λ was 0.689 Å and the camera length was 3 m.

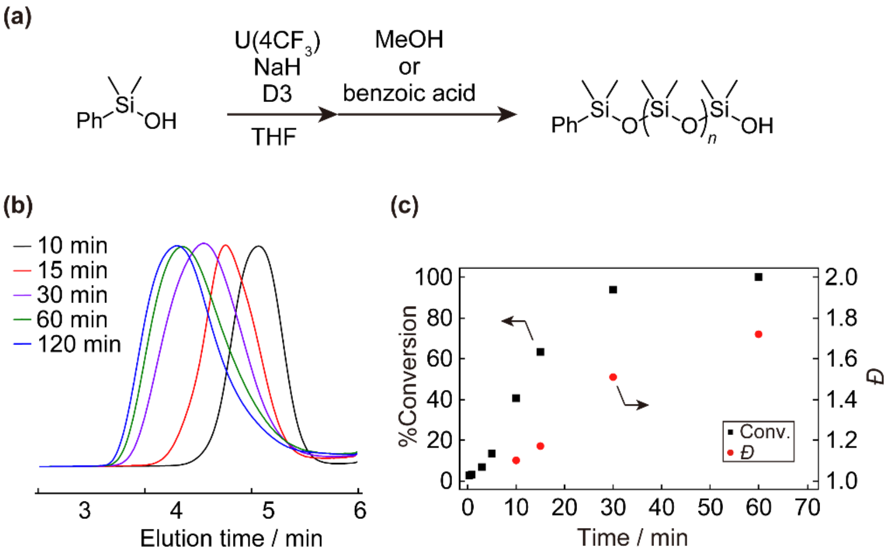

Figure S1. (a) ROP of D3 initiated from dimethylphenylsilanol catalyzed by urea(4CF$_3$) anion. (b) SEC traces of products after ROP of D3 with the polymerization time of 10, 15, 30, 60, and 120 min, respectively. (c) Plots of conversion and *Đ* against time.

Scheme S1. Synthetic route for PRNs.

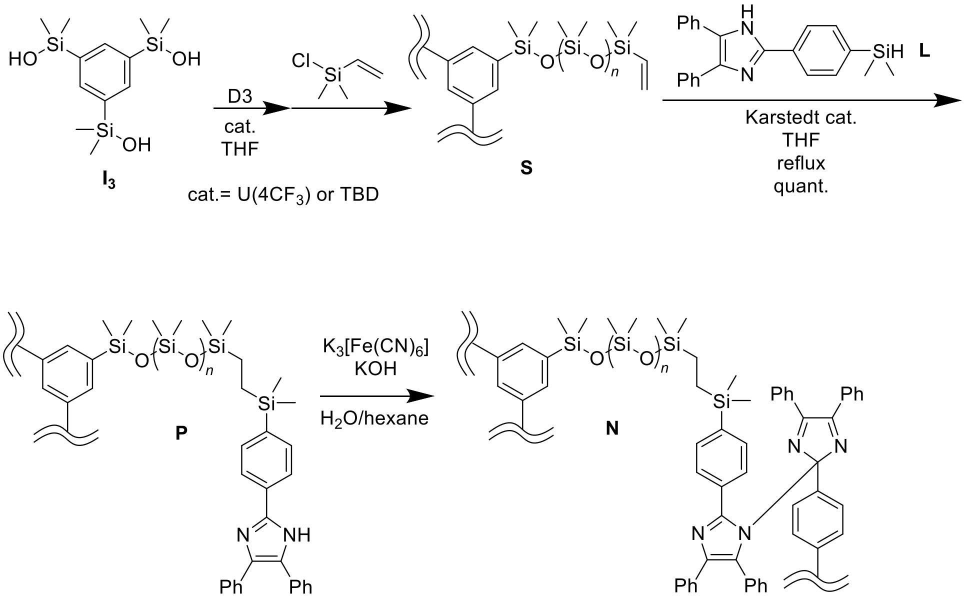

Table S1. ROP of D3 initiated from $\mathbf{I_3}$.

Entry	Catalyst	$[M]_0/[\mathbf{I_3}]_0$	Time (min)	Conv. (%)[a]	M_n[b]	M_w[c]	$Đ$[d]
1[e]	$U(4CF_3)/NaH$	270	10	48	6800	7200	1.05
2[e]	$U(4CF_3)/NaH$	270	30	83	9200	10300	1.12
3[e]	$U(4CF_3)/NaH$	270	61	96	11000	13600	1.23
4[e]	$U(4CF_3)/NaH$	270	120	>99	13500	19300	1.43
5[f, g]	TBD	50	80	94	9200	12300	1.34
6[g, h]	TBD	167	450	73	50400	68200	1.35

[a]Conversion determined by ^1H NMR. [b]Number average molecular weight, determined by SEC with RI detector. [c]Weight average molecular weight, determined by SEC with RI detector. [d]Dispersity ($Đ = M_w/M_n$), determined by SEC. [e]$[\mathbf{I_3}]_0 : [NaH]_0 : [U(4CF_3)]_0 : [M]_0 = 1 : 9 : 9 : 270$, $[M]_0 = 2.4$ M. [f]$[\mathbf{I_3}]_0 : [TBD]_0 : [M]_0 = 1 : 1 : 50$, $[M]_0 = 2.3$ M. [g]SEC measurements were performed after terminating with chlorodimethylvinylsilane. [h]$[\mathbf{I_3}]_0 : [TBD]_0 : [M]_0 = 1 : 1 : 500$, $[M]_0 = 2.3$ M.

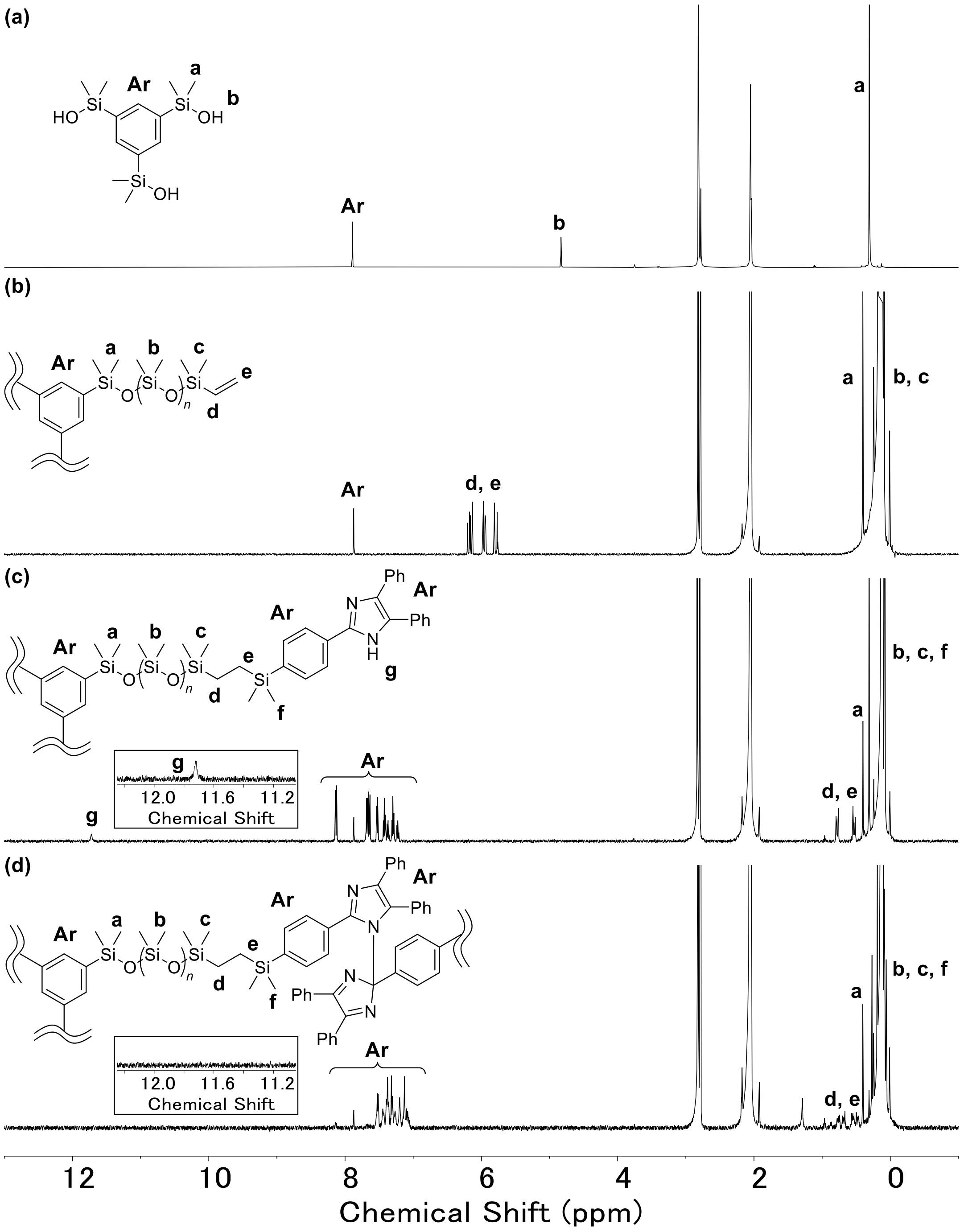

Figure S2. 500 MHz ^{1}H NMR spectra of (a) **I$_3$**, (b) **S$_1$**, (c) **P$_1$**, and (d) acetone soluble part of **N$_1$** (acetone-d_6). Insets are magnified ^{1}H NMR spectra around 11.2–12.2 ppm.

Table S2. SEC characterization of star-shaped PDMSs.

Polymers	M_n[a]	M_w[b]	M_p[c]	$Đ$[d]
$\mathbf{S_1}$	6600	6900	6700	1.05
$\mathbf{S_2}$	9200	12300	11200	1.34
$\mathbf{S_3}$	50400	68200	63300	1.35
$\mathbf{P_1}$	7500	7900	7500	1.06
$\mathbf{P_2}$	8900	13000	11400	1.47
$\mathbf{P_3}$	42300	66200	50700	1.56

[a]Number average molecular weight, determined by SEC with RI detector. [b]Weight average molecular weight, determined by SEC with RI detector. [c]Peak molecular weight, determined by SEC with RI detector. [d]Dispersity (= M_w/M_n), determined by SEC.

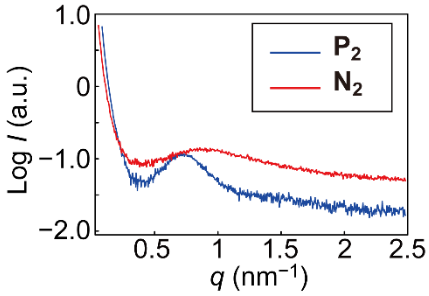

Figure S3. SAXS profiles of $\mathbf{P_2}$ and $\mathbf{N_2}$ recorded at BL-10C in KEK-PF.

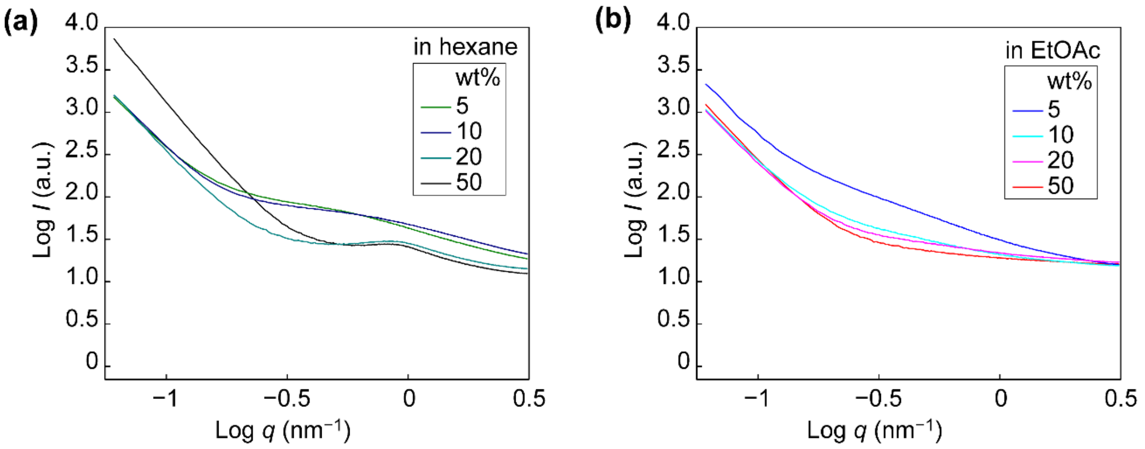

Figure S4. Concentration-dependent SAXS profiles of $\mathbf{N_2}$ swollen with (a) hexane and (b) EtOAc recorded at BL19B2 in SPring-8.

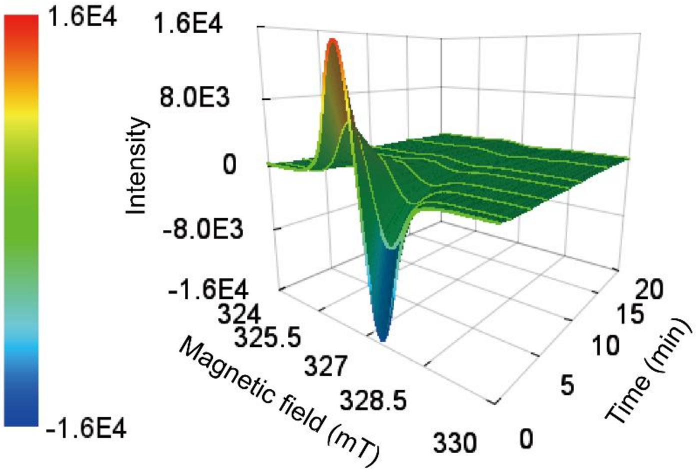

Figure S5. Three-dimensional stacked plots of ESR spectra over time. The **N_1** was irradiated with light (λ ~ 410 nm) and the measurements were started immediately after terminating irradiation.

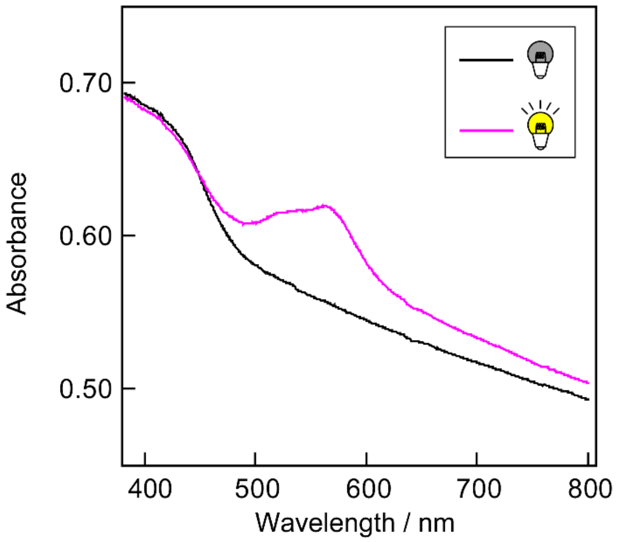

Figure S6. UV-vis spectra of a thin film formed from **N_1** without (black line) and with (pink line) photoirradiation (λ = 365 nm).

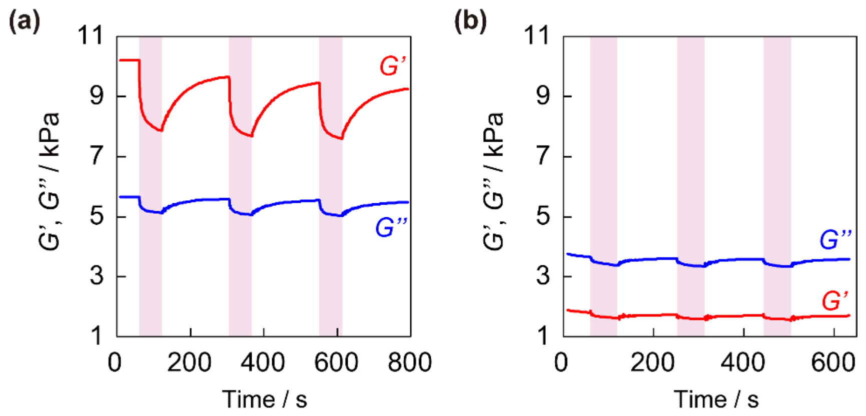

Figure S7. Time-course plots of *G'* and *G''* upon ON–OFF of UV irradiation to (a) $\mathbf{N_2}$ and (b) $\mathbf{N_3}$. Photoirradiation (λ = 365 nm) was performed during the region indicated with pink.

References

[1] S. Honda, M. Oka, H. Takagi, T. Toyota, *Angew. Chem. Int. Ed.* **2019**, *58*, 144.
[2] B. Lin, R. M. Waymouth, *J. Am. Chem. Soc.* **2017**, *139*, 1645.
